# A prospective trial to evaluate the clinical efficacy and safety of neoadjuvant chemotherapy with arsenic trioxide and carboplatin in locally advanced cervical cancer: a study protocol for randomized controlled clinical

**DOI:** 10.1186/s13063-022-06489-1

**Published:** 2022-07-08

**Authors:** Ze Yang He, Hai Ying Li, Jiao Yan, Si Jin Li, Dao Cheng Li, Zhong Zhe Liang

**Affiliations:** Department of Gynecology, The First Affiliated Hospital of Guangzhou University of Chinese Medicine, Guangzhou, China

**Keywords:** Cervical cancer, Arsenic trioxide (AS_2_O_3_), Carboplatin, Paclitaxel, Protocol, Randomized controlled trial, Neoadjuvant chemotherapy

## Abstract

**Background:**

Cervical cancer is the fourth most common malignancy in women, which is threatening female reproductive tract health. Chemotherapy can be used for neoadjuvant therapy of locally advanced cervical cancer and postoperative adjuvant therapy for patients with high-risk factors, so as to reduce the focus, sensitize radiotherapy, and reduce recurrence. The current first-line treatment is paclitaxel combined with platinum. Many literature studies have found that As_2_O_3_ alone or in combination with platinum drugs have good efficacy in a variety of tumors both in vivo and in vitro. Moreover, our research group has verified that the efficacy of As_2_O_3_ combined with platinum drugs in the treatment of cervical cancer is not inferior to the traditional first-line regimen at the cellular and animal levels, and paclitaxel is more expensive than As_2_O_3_. Hence, we aim to evaluate the clinical efficacy and safety of neoadjuvant chemotherapy with As_2_O_3_ and carboplatin in locally advanced cervical cancer.

**Methods:**

Sixty participants in the IB2, IIA2, and IIB stages of cervical cancer will be recruited in this study. After excluding patients who did not meet the criteria, they were randomly assigned to two groups in a 1:1 ratio. All patients underwent colposcopic biopsies to confirm the diagnosis and detailed clinical examinations. Eligible patients will receive either 2 cycles of paclitaxel and carboplatin or As_2_O_3_ and carboplatin every 3 weeks. Patients were assessed for clinical efficacy after the second cycle of chemotherapy. Patients who had disease stable or disease progression at these time points will receive concurrent chemotherapy and radiation directly, while responders will receive PiverRutledge grade III radical hysterectomy and bilateral pelvic lymphadenectomy. Both groups of patients undergoing radical hysterectomy were given adjuvant therapy as per protocol-defined criteria. The efficacy and toxicity of the two groups were evaluated according to WHO acute and subacute toxicity classification standards.

**Discussion:**

This is the first single-center, prospective, two-arm design, open-label randomized control trial that will evaluate the clinical efficacy and safety of neoadjuvant chemotherapy with As_2_O_3_ and carboplatin in locally advanced cervical cancer.

**Trial registration:**

ChineseClinicalTrialRegistryChiCTR1900023822. Registered on 13 June 2019.

**Supplementary Information:**

The online version contains supplementary material available at 10.1186/s13063-022-06489-1.

## Background

Up to now, it is estimated that there are 19.3 million new cases and 10 million cancer deaths in the world, and half of the cancer cases and cancer deaths occur in Asia. Among them, 604,127 new cases and 341,831 deaths occur in cervical cancer, ninth among 36 types of cancer and fourth among female malignant tumors [1].

According to the International Union of Obstetrics and Gynecology (FIGO) 2009 staging, the standard treatment for patients with locally advanced cervical cancer, namely stage IB2–IVA, is platinum-based concurrent chemotherapy and radiotherapy [2, 3].

Chemotherapy plays an important role in locally advanced cervical cancer and can be used for neoadjuvant therapy of locally advanced cervical cancer and postoperative adjuvant therapy of patients with high-risk factors, so as to achieve the purpose of shrinking lesions, sensitizing radiotherapy, reducing recurrence, and so on. Neoadjuvant chemotherapy is to reduce tumor volume and kills small lesions through chemotherapy; facilitates subsequent surgery, radiotherapy, and other treatments; and brings new possibilities for patients with locally advanced cervical cancer. Many past studies have found that surgical or radiotherapy after neoadjuvant chemotherapy seems to have better survival outcomes than direct chemoradiotherapy [4–7]. However, it has recently been found that compared with direct chemoradiotherapy, radiotherapy after neoadjuvant chemotherapy is less effective and more prone to some toxic and side reactions [8]. A phase III clinical trial from a single center found that platinum-based concurrent chemoradiotherapy for locally advanced cervical cancer achieved better DFS than radical surgery after neoadjuvant chemotherapy, and OS was comparable [9]. So, neoadjuvant chemotherapy plus surgery is still very controversial and lacks sufficient evidence.

Arsenic trioxide (As_2_O_3_) is one of the active components of traditional Chinese medicine arsenic. Since the 1970s, Chinese experts have applied it to patients with acute promyelocytic leukemia (APL) and found that its remission rate reaches about 80%, and its toxic side effects such as bone marrow suppression are small, so it has been recognized and widely used worldwide. In 2001, it was approved by the American Food and Drug Administration (FDA) for the treatment of APL by fast track [10]. Later studies at home and abroad have found that As_2_O_3_ has a complex and extensive tumor inhibition mechanism, and its therapeutic effect has been extended to breast cancer [11], lung cancer [12, 13], gastric cancer [14, 15], liver cancer [16, 17], bladder cancer [18], cervical cancer [19], and other solid tumors. In the field of cervical cancer research, the tumor inhibition mechanism of As_2_O_3_ mainly focuses on the following aspects: (1) affect the proliferation and apoptosis of cervical cancer cells (interfere with the tumor cell cycle, affect the expression of genes related to proliferation and apoptosis, and affect the intracellular structure and function); (2) prevent the formation of tumor blood vessels and the invasion and metastasis of cervical cancer cells; (3) enhanced radiosensitivity; and (4) tumor immunosuppression was downregulated [20–26].

Based on a large number of the previous cell and animal experiments and clinical data on the combined use of As_2_O_3_ and carboplatin, this study is the first to use As_2_O_3_ combined with carboplatin in patients with cervical cancer, which has good operability and safety, and to explore an effective, safe, and economic drug for cervical cancer.

## Materials and methods

### Objective

The primary objective of this study is to evaluate the efficacy of the As_2_O_3_ plus carboplatin regimen as neoadjuvant chemotherapy for cervical cancer. The secondary objective of this study is to evaluate the safety of the As_2_O_3_ plus carboplatin regimen as neoadjuvant chemotherapy for cervix cancer.

### Study design

**Fig. 1:**
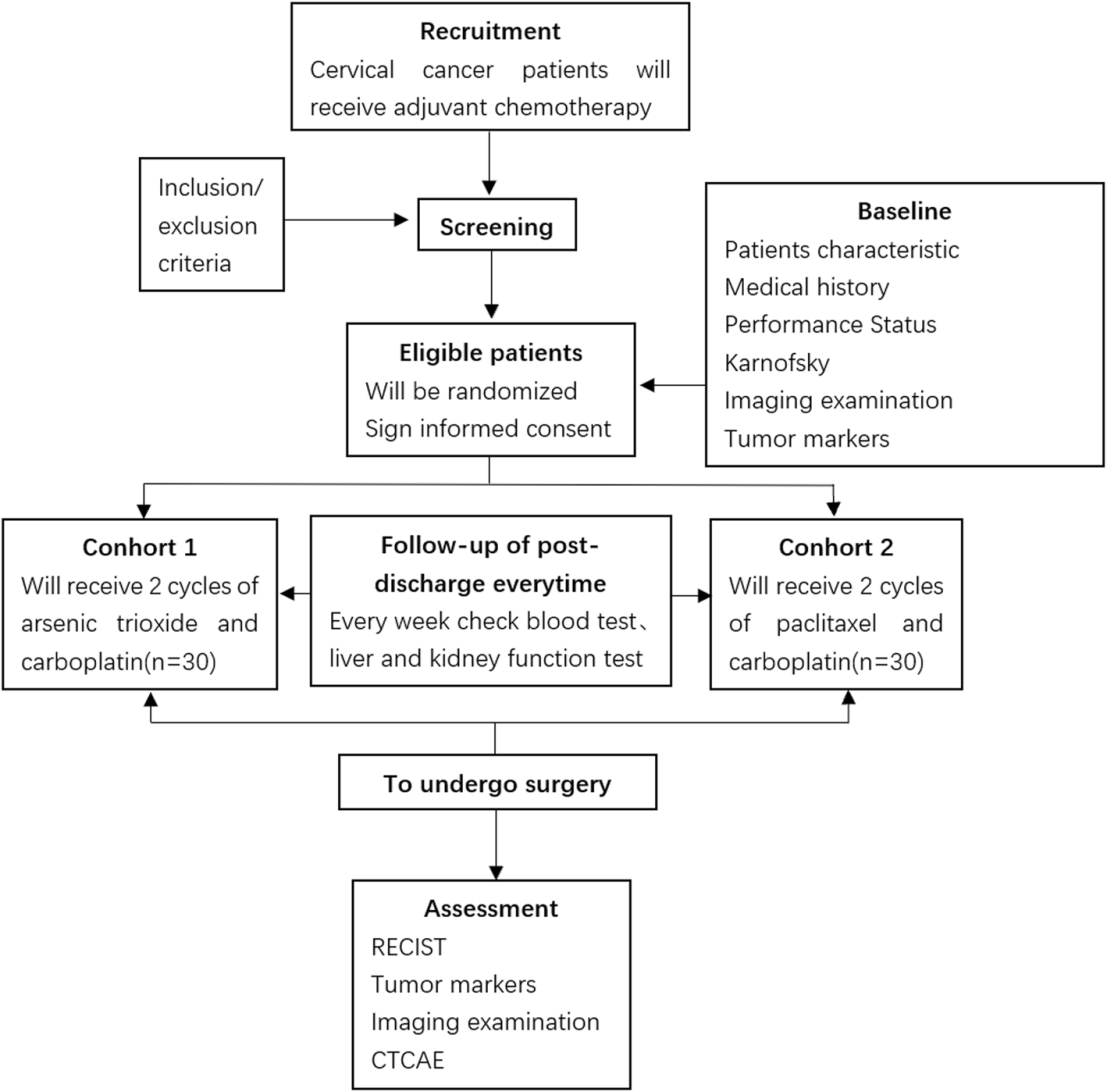
Flow chart of the study. RECIST, Response Evaluation Criteria in Solid Tumors (version 1.1); CTCAE, Common Terminology Criteria for Adverse Events (version 4.03)

This trial is a single-center, non-inferiority, controlled, randomized, prospective study. The study started in January 2019 and is still ongoing. We recruited histopathologically confirmed patients with IB2–IIB stage cervical cancer without distant metastasis from the First Affiliated Hospital of Guangzhou University of Chinese Medicine according to the cervical cancer FIGO staging (2009). Patients with cervical cancer will be randomly allocated to either the experimental group or the control group, then they will be administrated As_2_O_3_ plus carboplatin or paclitaxel plus carboplatin for two cycles, which may last up to 6 weeks. Participants will be followed until surgery or drop out of the study. An overview of the study is shown in Fig. 1. The Standard Protocol Items: Interventional Trial Recommendations (SPIRIT) Checklist is used to detail the study protocol [27] (see Table 1). Two supervisors will monitor the accuracy and feasibility of the process. The institutional ethics review board approved the study protocol.

**Table 1:** Example template of recommended content for the schedule of enrollment, interventions, and assessments

Time point	Screening	Adjuvant chemotherapy				Close-out
	− t1–0	t1	t2	t3	t4	t5–tx
Enrollment						
Screening	X					
Informed consent	X					
Program						
Cohort 1 (ATO + P)	X	X	X	X	X	X
Cohort 1 (T + P)	X	X	X	X	X	X
Assessment						
Patients characteristic	X					
Medical history	X					
Performance status	X					
Karnofsky	X					
Blood test		X	X	X	X	X
Liver function test		X	X	X	X	X
Kidney function test		X	X	X	X	X
Color Doppler ultrasound		X		X		X
Tumor markers		X		X		X
Imaging examination (MRI)		X				X
RECIST						X
CTCAE						X
Cohort migration						X
Combined medication						X

Imaging examination contains magnetic resonance imaging (MRI) or computed tomography (CT); tumor markers include the following: *AFP* alpha-fetoprotein antigen, *CEA* carcinoembryonic antigen, *CA153* cancer antigen 153, *CA199* cancer antigen 199, *CA125* cancer antigen 125, *SCC* squamous cell carcinoma antigen, *CTCAE* Common Terminology Criteria for Adverse Events, *ATO* arsenic trioxide, *T* Taxol, *C* carboplatin

### Inclusion criteria

Participants must meet the following inclusive criteria: (1) age 20–60; (2) histopathologically proven stage IB2, IIA2, IIB cervical carcinoma without therapy; (3) the Karnofsky Functional Status Score should be at least 70; (4) blood test and liver and kidney function were normal before treatment; (5) there was no evidence of distant metastasis; and (6) no serious heart, liver, kidney, blood system, and other important organ diseases.

### Exclusion criteria

Exclusion criteria are as follows: (1) there is recurrence or distant metastasis; (2) prior radiotherapy, chemotherapy, immunotherapy, or surgery; (3) serious comorbidities, pregnancy, or lactation; (4) allergic to arsenic and participated in other clinical trials in the past 3 months; and (5) the condition is too late to receive surgery combined with adjuvant chemotherapy.

### Setting and participants

#### Participant recruitment and screening

The recruitment process of subjects will be publicized by following the WeChat official public account at the Gynecology Department of the First Affiliated Hospital of Guangzhou University of Chinese Medicine, and in cooperation with the Oncology Department of our hospital, cervical cancer patients with surgical indications will be transferred to the cervical cancer research team, and our department experts with oncology background will be screened. Qualified candidates will be invited to participate in the selection process. All patients underwent colposcopic biopsies to confirm the diagnosis and detailed clinical examinations, including pelvic examinations by two clinicians to determine the initial stage. Patients also received blood tests, color ultrasound of computed tomography, or magnetic resonance imaging of the abdomen and pelvis before being randomly assigned.

#### Study protocol

Eligible patients will receive either 2 cycles of paclitaxel (135–175 mg/m^2^, day 1) and carboplatin (dose 5 to 6 zones below the curve, day 1) or As_2_O_3_ (8 mg/m^2^, days 1 to 7) and carboplatin (dose 5 to 6 zones below the curve, day 1) every 3 weeks. Patients were assessed for clinical efficacy after the second cycle of chemotherapy. Patients who had disease stable or disease progression at these time points will receive concurrent chemotherapy and radiation directly, while responders will receive PiverRutledge grade III radical hysterectomy, bilateral pelvic lymphadenectomy, and, if necessary, paraaortic subaortic lymph node sampling by a specialist gynecologic oncologist 3 weeks after the second cycle of chemotherapy.

#### Follow-up treatment

According to the NCCN guidelines, both groups of patients undergoing radical hysterectomy were given adjuvant therapy (radiotherapy or concurrent chemotherapy and radiotherapy) as per protocol defined [28]. According to the histopathological evaluation of surgical specimens, adjuvant chemoradiotherapy was given in the case of positive surgical margin, lymph node metastasis, or parametrium involvement. Based on any two of the following characteristics: deep cervical interstitial invasion, tumor size > 4 cm, or lymphatic vascular invasion, adjuvant radiotherapy was given alone. The efficacy and toxicity of the two groups were evaluated according to WHO acute and subacute toxicity classification standards (RECIST).

### Randomization and blinding

Qualified patients were randomly assigned to either study group in a 1:1 ratio using a random number table. Treatment assignments were uncovered as follows: Once informed consent was obtained from eligible patients, the numbered, opaque, sealed envelopes containing treatment details were opened by the investigators, and patients were assigned corresponding interventions.

### Sample size

The main aim of this research was to study the efficacy of neoadjuvant chemotherapy for cervical cancer and to use this as the basis for sample size calculation. Based on a related large cohort study [5], the effective rate in patients with locally advanced cervical cancer was approximately 67%. The G-power (V.3.1) formula was used to calculate the sample size difference between the two independent ratios in non-inferiority trials. Power calculations assumed 1:1 randomization, a two-sided test with a type I error rate (*α*) of 0.05, and a type II error rate (1−*β*) of 0.8. The non-inferiority margin for the primary outcome was set at 0.9 by the trial clinicians and statisticians. We want 25 participants per queue. Considering the 20% loss-to-follow-up rate, the sample size should be 30 per cohort and 60 overall.

### Outcome

An investigator will be put in charge of the following up weekly by telephone or outpatient.

#### Primary outcome

The study’s primary outcome is the effective rate of NACT, that is to say, how much the tumor shrinks after curative treatment. At the end of the study, this indicator will be calculated by the response evaluation criteria in solid tumors [29] (RECIST) The results in the assessment of tumor size will be obtained by computed tomography or magnetic resonance imaging. Results assessment of tumor markers will be obtained by blood drawn. Unless follow-up is lost, the date of the last assessment will be recorded after two cycles of the NACT.

#### Secondary outcomes

The secondary outcome of this study is the safety of NACT, which is evaluated by adverse events (AE) and adverse drug reactions (ADR). AE refers to adverse medical events that occur after patients or clinical trial subjects receive a drug, but they do not necessarily have a causal relationship with the treatment. ADR refers to the harmful, but not expected, causal reaction that occurs during the normal application of the drug at the prescribed dose. To elucidate AE and ADR, we will evaluate the following secondary outcomes: blood analysis, coagulation function, hepatic and renal function, and gastrointestinal adverse events. The toxic effects of patients during chemotherapy were defined accordingly to NCI-CTCAE (v. 4.03) of grade 3 and above [30]. The clinical research will develop a corresponding data security monitoring plan based on the level of risk. All adverse events are recorded in detail, properly handled, and tracked until they are properly resolved or the condition is stable, and report serious adverse events and unexpected events to the ethics committee, competent authorities, sponsors, and drug regulatory authorities promptly under regulations; the main investigator regularly checks all adverse events, which shall be reviewed cumulatively, and researchers’ meetings shall be held when necessary to evaluate the risks and benefits of the research; researches with greater than the minimum risk will be arranged with independent data monitors to monitor the research data, and high-risk research will establish independent data to ensure security, and the Supervisory Committee monitors the accumulated safety data and effectiveness data to make recommendations on whether the study should continue.

### Statistical analysis

Statistical analysis will be conducted using the SPSS software V.24.0 (SPSS Inc., Chicago, IL, USA). The *χ*^2^ test will be used for counting the data of the two groups. Measurement data will be expressed as mean ± standard deviation (‾*x* ± *s*), and a *T*-test will be used, too. *P*-value < 0.05 means the difference is statistically significant.

## Discussion

Stage IB2–IVA cervical cancer is treated with chemoradiotherapy, surgery, or both. However, if the tumor is too large or too deep, or too wide, it will add difficulty to the surgical treatment. Chemotherapy alone can eliminate many small lesions but has relatively little effect on large masses, and radical radiotherapy, including external beam radiation and endoluminal therapy, can provide a good chance of being cured, but the maximum radiation dose given to patients is limited by the tolerance of normal tissues giving, especially adjacent tissues that are prone to radiation proctitis, radiation bladder side effects such as inflammation, greatly reduce the quality of life of patients, and the cost of radiotherapy is expensive. If the small metastatic lesions are controlled by chemotherapy first and the tumor size is reduced, the chance of surgery can be greatly increased, even the difficulty of surgery can be reduced, and the burden of adjuvant therapy can be reduced. Therefore, neoadjuvant chemotherapy seems to be able to ensure both effectiveness and achieve certain economic effects.

As_2_O_3_ is now commonly used in the treatment of acute promyelocytic leukemia, and its low cost, side effects, and a wide range of anti-tumor effects. The best example of its application in other solid tumors is liver cancer, which has shown good efficacy in both single and combination drugs, in vivo and in vitro, and even in clinical trials. Therefore, in 2004, China’s Food and Drug Administration approved As_2_O_3_ for advanced liver cancer [31]. There have been many studies trying to combine As_2_O_3_ with platinum and other first-line chemotherapy regimens to observe its efficacy increases in various solid tumors, and whether patients tolerate side effects: there are reports of As_2_O_3_ combined with ICE regimen (ifosfamide + cisplatin + etoposide) in the treatment of relapsed and refractory non-Hodgkin’s lymphoma with a clinical total effective rate 60%. The main adverse reactions were bone marrow suppression, and leukopenia of grade III~IV was 12/15 (80.0%). However, patients can tolerate it, so the efficacy is good and side effects can be tolerated, which is worthy of clinical promotion [32]. Compared with cisplatin alone, As_2_O_3_ combined with intracavitary injection in the treatment of malignant pleural effusion has increased efficacy and similar adverse reactions, which can be tolerated by patients [33]. Reviewing the cases of extremity osteosarcoma lung metastases in the Bone and Soft Tissue Tumor Center of Peking University People’s Hospital for more than 10 years, it was found that As_2_O_3_ combined with first-line chemotherapy (doxorubicin, cisplatin, high-dose methotrexate, and ifosfamide) may be an effective and tolerant new treatment option [34]. As_2_O_3_ combined with cisplatin showed good efficacy in the treatment of advanced gallbladder cancer, with few adverse reactions, mainly digestive tract reactions, mild bone marrow suppression, and liver function impairment [35]. How about applying it to cervical cancer? In animal experiments, the combined use of As_2_O_3_ and cisplatin increased the efficacy of the single drug on the transplanted tumor of cervical cancer in nude mice, and the examination of blood and the dissection of liver and kidney organs in nude mice showed no increase in bone marrow suppression, liver and kidney injury, and other toxic and side effects [36]. In addition, it has been reported that As_2_O_3_ has been used in the clinical application of cervical cancer. When As_2_O_3_ is used in patients with cervical cancer radiotherapy, it is found that its sensitization effect on cervical cancer radiotherapy is similar to that of cisplatin, but its side effects are smaller [37]. Therefore, what about combining As_2_O_3_ with platinum drugs for neoadjuvant chemotherapy? And can it be non-inferior to the standard first-line chemotherapy regimen of paclitaxel combined with platinum?

Of course, our study also has some limitations. Firstly, only patients with stages IB2, IIA2, and IIB were included. To be able to give full play to maximum advantages of the neoadjuvant chemotherapy combined surgery, cases of stage III and IVA surgery may be more difficult to operate on. Owing to the small lesion of stage IIA1, direct chemoradiotherapy or direct postoperative adjuvant therapy is more effective, while for stages IB2, IIA2, and IIB, due to the large size of the tumor, direct surgery may be more difficult, and direct radiotherapy can greatly stimulate adjacent organs on account of the dose. At this time, neoadjuvant chemotherapy combined with surgery can be used to achieve ideal results. Secondly, as this study was a single-center study with limited staging, it was relatively difficult to collect cases and the number of cases was greatly reduced. Therefore, under the condition of minimizing bias, the number of cases included in this study was small, but if the results are ideal, the study scale can be expanded. Thirdly, because the As_2_O_3_ solution needs to be used continuously for 8 days, while the other one needs only one day, double-blind method could not be realized in this study, which may bring some selective bias.

This proposal is mainly to observe the efficacy and safety of arsenic trioxide combined with platinum drugs in neoadjuvant chemotherapy. The results of this study will provide a new possibility for the treatment options for cervical cancer.

## Trial status

Participant recruitment started in January 2019 and is still ongoing.

## Supplementary Information

**Additional file 1. **SPIRIT 2013 Checklist. Recommended items to address in a clinical trial protocol and related documents.

## Data Availability

Datasets obtained during this study can be provided by the corresponding author upon reasonable request.

## Abbreviations

AS_2_O_3_/ATO: Arsenic trioxide
FIGO: International Union of Obstetrics and Gynecology
APL: Acute promyelocytic leukemia
FDA: American Food and Drug Administration
RECIST: Response Evaluation Criteria in Solid Tumors
NACT: Neoadjuvant chemotherapy
AE: Adverse events
ADR: Adverse drug reaction
CTCAE: Common Terminology Criteria for Adverse Events
ICE: Ifosfamide + cisplatin + etoposide
